# Supplementary material for: Characterization of DNA methylation in Malawian Mycobacterium tuberculosis clinical isolates
Source: PeerJ. 2020 Dec 16;8:e10432. doi: 10.7717/peerj.10432 (PMC7749660; doi:10.7717/peerj.10432)
Supplement: Supplemental Information 1 [file peerj-08-10432-s001.pdf]

Supporting Table 1: Methylation efficiency for 34 Mycobacterium tuberculosis samples

Sample	Lineage	CACGCAG 820)	CTCCAG (1947)	CTGGAG (1947)	GATNNNNRTAC (363)	GTAYNNNNATC (363)
ERS2711939	L4	0.96585363	0.972265	0.94966614	0	0
ERS2711940	L4	0.9792683	0.97842836	0.9686698	0	0
ERS2711941	L4	0.96707314	0	0.58294815	0	0
ERS2711942	L4	0.9768293	0.98151004	0.96712893	0	0
ERS2711943	L1	0.5609756	0.9866461	0.972265	0.93663913	0.93112946
ERS2711944	L4	0.96585363	0.9712378	0.94453007	0	0
ERS2711945	L4	0.7390244	0.7786338	0.68875194	0	0
ERS2711946	L4	0.96829265	0.97740114	0.95428866	0	0
ERS2711947	L4	0.97195125	0.9712378	0.9481253	0	0
ERS2711948	L1	0	0.5100154	0.43348742	0	0.3168044
ERS2711949	L4	0.95731705	0.9583975	0.9244992	0	0
ERS2711950	L4	0.97195125	0.9820236	0.9583975	0	0
ERS2711951	L4	0.9646341	0.9794556	0.95017976	0.90082645	0.8953168
ERS2711952	L4	0.9487805	0.96302	0.9342578	0	0
ERS2711953	L2	0.65	0	0	0.4903581	0.47933885
ERS2711954	L2	0.95609754	0	0	0.8787879	0.8595041
ERS2711955	L2	0.9634146	0	0	0.8787879	0.8815427
ERS2711956	L1	0	0.33538777	0.26759118	0	0
SAMEA104606019	L1	0.7963415	0.990755	0.99126863	0.969697	0.9641873
SAMEA104606020	L1	0.7134146	0.9902414	0.9892142	0.96694213	0.9614325
SAMEA104606021	L1	0.7487805	0.98767334	0.9871597	0.9641873	0.9586777
SAMEA104606022	L5	0.997561	0.97842836	0.972265	0.9889807	0.9889807
SAMEA104606023	L2	0.9902439	0	0	0.95592284	0.9614325
SAMEA104606024	L4	0.99512196	0.98870057	0.9851053	0	0
SAMEA104606025	L4	0.99268293	0.96302	0.96764255	0.94490355	0.95316803
SAMEA104606026	L6	0.9902439	0.978942	0.97791475	0.9476584	0.95592284
SAMEA104606027	L6	0.9890244	0	0	0.862259	0.8705234
SAMEA104606028	L2	0.9902439	0	0	0.92837465	0.93112946

Supporting Table 1: Methylation efficiency for 34 Mycobacterium tuberculosis samples

SAMEA104606029	L4	0.99512196	0.9892142	0.9861325	0	0
SAMEA104606030	L5	0.9890244	0.9650745	0.9619928	0.9834711	0.9807162
SAMEA104606031	L6	0.9890244	0.9820236	0.98099643	0.9614325	0.95592284
SAMEA104606032	L6	0.9890244	0.972265	0.97072417	0.94490355	0.9476584
SAMEA104606033	L6	0.99268293	0.9763739	0.9753467	0.95592284	0.9586777
SAMEA104606034	L6	0.9902439	0.96815616	0.9661017	0.9476584	0.94490355
